# Acute massive pulmonary embolism treated by urgent pulmonary embolectomy: A case report

**DOI:** 10.1002/ccr3.2913

**Published:** 2020-05-03

**Authors:** Tsuyoshi Tagawa, Shigeki Sakuraba

**Affiliations:** ^1^ Division of Clinical Anesthesia Mie University Hospital Mie Japan; ^2^ Department of Anesthesiology Clinical Care Medicine Kanagawa Dental University Kanagawa Japan

**Keywords:** acute pulmonary embolism, intravenous immunoglobulin, patent foramen ovale, pulmonary embolectomy

## Abstract

The management of acute massive pulmonary embolism presents a clinical challenge as currently there is no consensus for definitive treatment. Early decision‐making regarding surgical intervention is essential when the risk of mortality is high.

## Graphical abstract

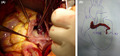

## 1 INTRODUCTION

Acute massive pulmonary embolism is a life‐threatening condition and must be immediately treated. The management of this condition typically presents a clinical challenge as currently there is no consensus for definitive treatment. Early decision‐making regarding surgical intervention is essential for successful management when the risk of mortality is high.

Acute massive pulmonary embolism (PE) has been continually associated with a high rate of mortality despite advances in diagnosis and therapy.^1^ The management of acute massive PE presents a clinical challenge as currently there is no established standard of care. Successful management strategies, primarily published in the form of case reports, include medical treatment along with cardiovascular support and anticoagulation, as well as more invasive interventions, such as pulmonary embolectomy. If acute massive PE is not diagnosed and treated early, it can cause hemodynamic deterioration, which warrants immediate surgery. Here, we describe the successful treatment of acute massive PE with pulmonary embolectomy.

## 2 CASE REPORT

**Figure 1:**
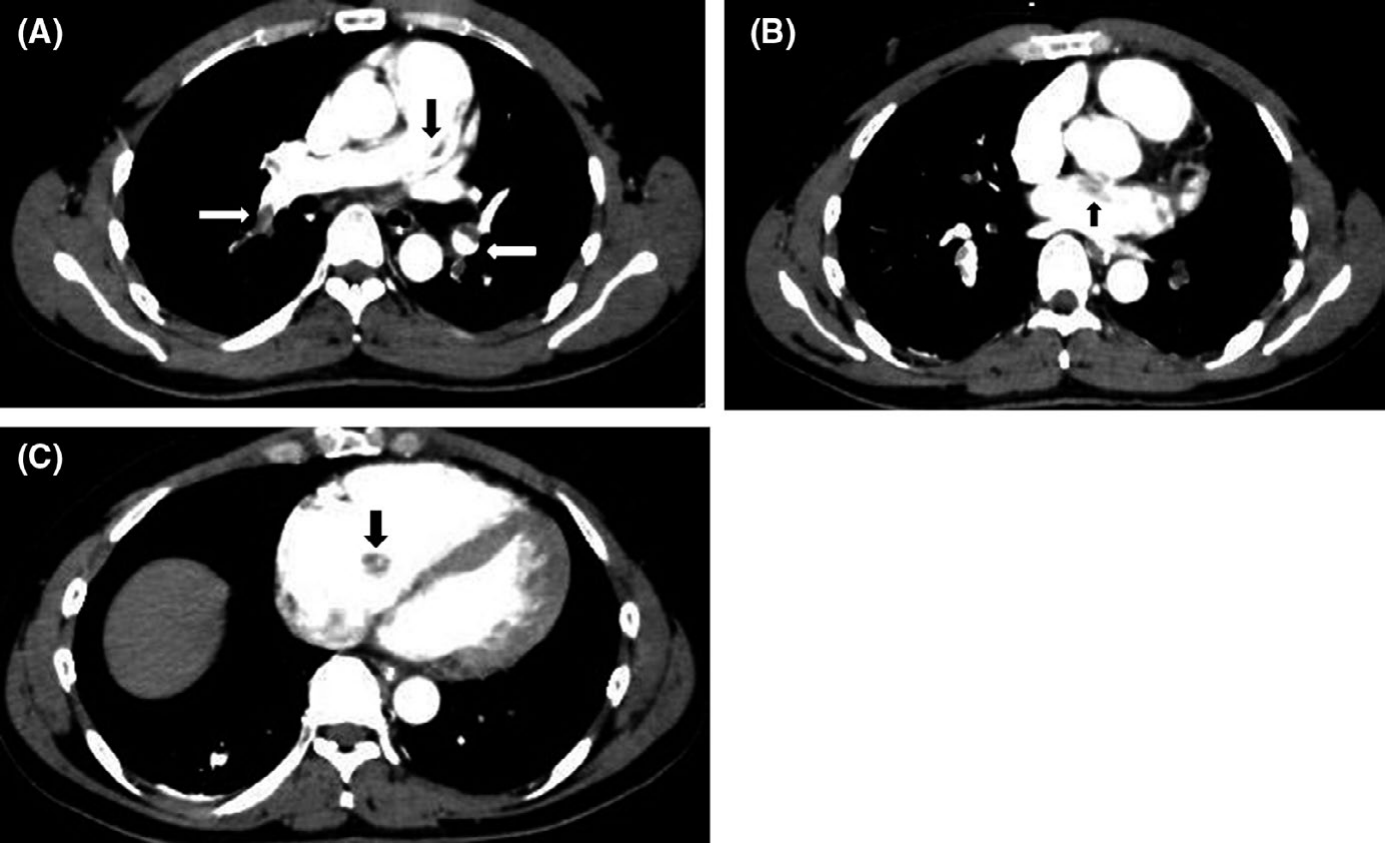
Preoperative chest computed tomography scan showing massive pulmonary emboli (A) and thrombi in the left (B) and right (C) atria (arrows)

A 37‐year‐old man with no medical history was admitted to a hospital with numbness in the arms and legs, as well as dysarthria and drowsiness. He was diagnosed with Bickerstaff brainstem encephalitis and treated with intravenous immunoglobulin (IVIG) for five days. He gradually recovered from coma and was nearly alert 2 weeks later. However, he suffered from sudden‐onset chest pain and abrupt reductions in blood pressure (BP) and oxygen saturation (SpO_2_). A chest computed tomography scan revealed massive emboli in both the lungs and thrombi in the bilateral atria (Figure 1). Consequently, heparin therapy was initiated and he was transferred to our institution for embolectomy. On arrival, he was alert and oriented with tetraparesis and dysarthria. His BP was 110/70 mm Hg, and heart rate was 120 beats/min. Arterial blood gas values with supplemental oxygen of 10 L/min administered via face mask were pH, 7.461; partial pressure of carbon dioxide, 43.6 mm Hg; and partial pressure of oxygen, 82.9 mm Hg. Transthoracic echocardiography revealed emboli in the pulmonary artery (PA) and bilateral atria; a right atrial embolus adherent to the tip of a central venous catheter (CVC) inserted from the femoral vein; and reduced blood flow in PA and consequent right ventricular dilatation, indicative of pulmonary hypertension, with an estimated ejection fraction of 59%.

**Figure 2:**
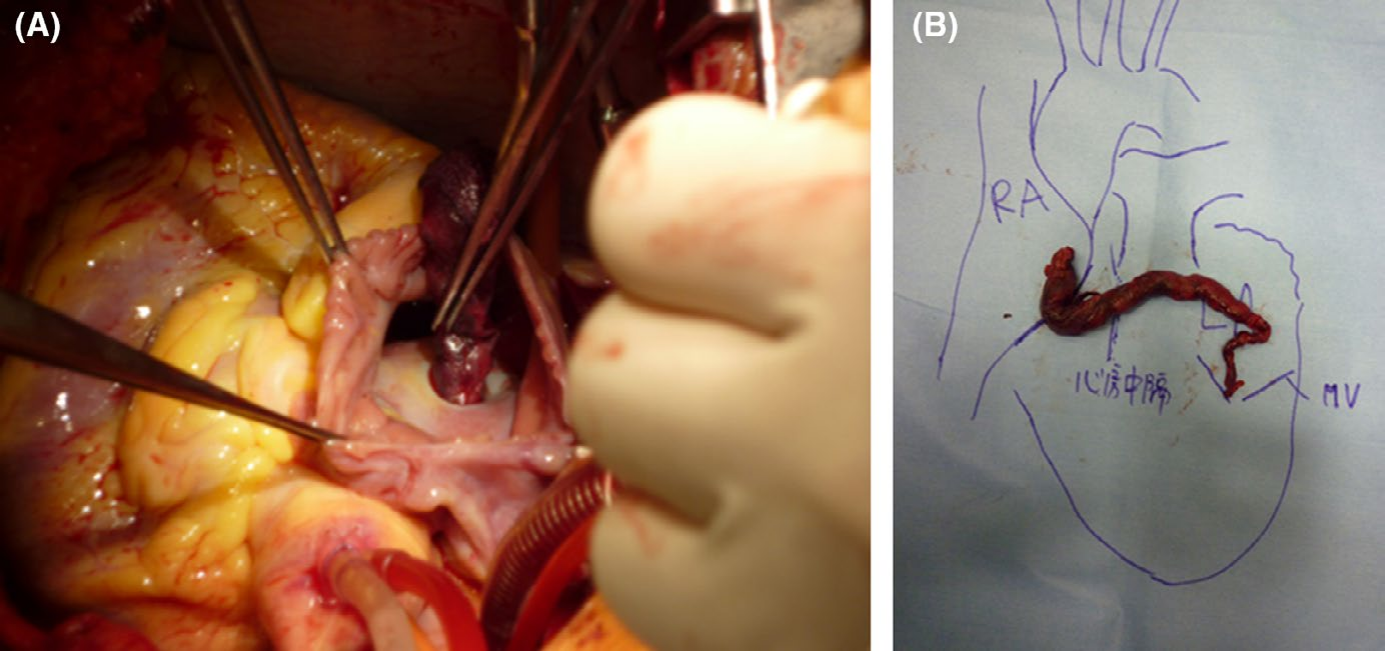
A, Thrombus extending from the right atrium through the patent foramen ovale to the left atrium. B, Rearranged thrombi extracted from bilateral atria

**Figure 3:**
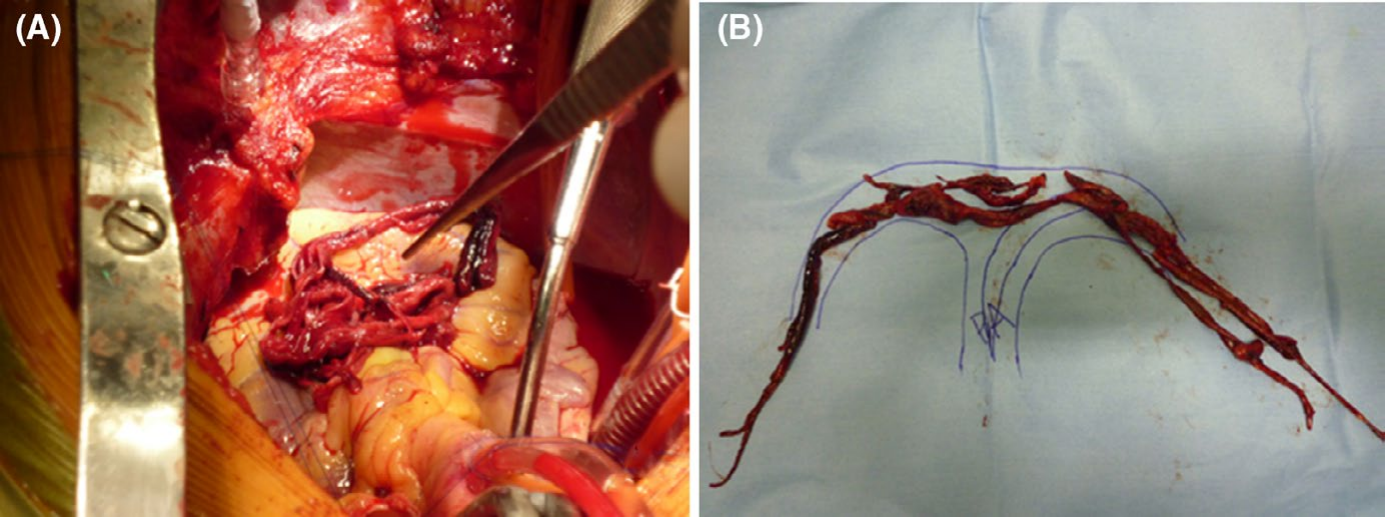
A, Thrombi extending from the main pulmonary artery to both the pulmonary arteries. B, Rearranged thrombi removed from both pulmonary arteries

Anesthesia was induced by administering fentanyl (50 μg), propofol (100 mg), and rocuronium (50 mg). The trachea was intubated, and anesthesia was maintained with fentanyl and sevoflurane. After intubation, BP decreased without alteration in SpO_2_. Intravenous ephedrine and rapid volume infusion restored BP. A Swan‐Ganz catheter was placed at the level of the right internal jugular vein to avoid dislocation of the thrombi. After median sternotomy and pericardiotomy, the patient was heparinized and cannulated for cardiopulmonary bypass (CPB). An arterial cannula was placed in the femoral artery, and a superior vena cava cannula was passed through the right atrium (RA). Total CPB was initiated by placing tapes around the superior and inferior venae cavae. The ascending aorta was cross‐clamped. An incision was made in RA, which exposed a thrombus extending from RA through the patent foramen ovale (PFO) to the left atrium; the thrombus was removed en bloc under direct vision using forceps (Figure 2). After ensuring the absence of residual thrombi in the left atrium, PFO was closed. Next, main PA was opened and thrombi that extended to both PAs were extracted in a manner similar to that used for extracting the thrombus in RA (Figure 3). After completing embolectomy, the Swan‐Ganz catheter was introduced into right PA and CVC was removed. RA was again accessed to ensure that no additional embolic material entered from the inferior vena cava; RA was then closed. After assuring complete clot removal using transesophageal echocardiography, separation from CPB was achieved with an infusion of dopamine (5 μg/kg/min). Simultaneously, an infusion of prostaglandin E_1_ (0.03 μg/kg/min) was also initiated based on the possibility of pulmonary vasoconstriction.

Systemic anticoagulation treatment was immediately initiated after the surgery. On postoperative day 1, the patient was extubated. On postoperative day 10, IVIG was restarted. He regained the ability to walk and, following an uneventful recovery, was transferred to a regional hospital for neurological rehabilitation on postoperative day 40. He had maintained adequate health for 12 months with continued oral warfarin therapy.

## 3 DISCUSSION

The prevalence of PE in hospitalized patients is approximately 1%.^2^, ^3^ Despite the current emphasis on deep venous thrombus prophylaxis, there is a failure rate of approximately 30%‐50%.^4^ The risk factors for PE include multiple trauma, pregnancy, cancer, heart failure, chronic deep venous insufficiency or prior venous thrombosis, long‐bone fractures, prolonged bed rest, obesity, and oral contraceptive use.^5^ In addition, IVIG therapy is associated with thromboembolic complications including PE.^6^, ^7^, ^8^ In the present case, IVIG in combination with immobilization and the presence of an indwelling CVC were presumed to have induced the formation of thrombi.

Although the treatment of massive PE remains controversial, there are two primary treatment modalities: thrombolytic treatment and pulmonary embolectomy. Thrombolysis is often effective but is associated with a high frequency of major bleeding complications, particularly intracranial hemorrhage.^1^, ^9^, ^10^ Previously, pulmonary embolectomy was reserved for patients with massive PE and severe hemodynamic instability because of its association with high mortality rate. Recently, some clinicians have reported a high survival rate of approximately 90% among patients with PE treated with pulmonary embolectomy, which was attributed to improved surgical techniques, rapid diagnosis and triage, and careful patient selection.^11^, ^12^ Although selected hemodynamically stable patients with PE have been treated without CPB and have shown good results,^13^, ^14^, ^15^, ^16^ we performed embolectomy with CPB owing to thrombi in cardiac chambers. Goldhaber reported a more than 10‐fold increase in mortality risk in individuals with PFO and an approximately 50% mortality rate if free‐floating, right‐side heart thrombi were observed during echocardiography.^17^ We believe that early decision‐making regarding surgical intervention was essential for successful management in the present case because the risk of death was sufficient to warrant immediate surgical correction.

## CONFLICT OF INTEREST

None declared.

## AUTHOR CONTRIBUTIONS

TT: drafted the manuscript, approved the manuscript, and collected the data. SS contributed to the critical revision and approval of the manuscript.
